# Profile of drugs used for self-medication by elderly attended at a referral center

**DOI:** 10.31744/einstein_journal/2018AO4372

**Published:** 2018-11-29

**Authors:** Samanta Bárbara Vieira de Oliveira, Soraya Coelho Costa Barroso, Maria Aparecida Camargos Bicalho, Adriano Max Moreira Reis

**Affiliations:** 1 Instituto Jenny de Andrade Faria de Atenção à Saúde do Idoso e da Mulher, Hospital das Clínicas, Universidade Federal de Minas Gerais, Belo Horizonte, MG, Brazil.; 2 Faculdade de Medicina, Universidade Federal de Minas Gerais, Belo Horizonte, MG, Brazil.; 3 Faculdade de Farmácia, Universidade Federal de Minas Gerais, Belo Horizonte, MG, Brazil.

**Keywords:** Aged, Self medication, Drug therapy, Drug-related side effects and adverse reactions, Idoso, Automedicação, Tratamento farmacológico, Efeitos colaterais e reações adversas relacionados a medicamentos

## Abstract

**Objective:**

To determine the profile of medications used for self-medication by the elderly.

**Methods:**

A cross-sectional study based on interviews with elderly seen at a reference center for Elderly Health of a teaching hospital, from July 2014 to July 2015. Clinical, demographic and pharmacotherapeutic data were collected.

**Results:**

A total of 170 elderly were interviewed, 85.9% female, and the median age was 76 years. The frequency of self-medication was 80.5%. The most used medications for self-medication were central acting muscle relaxants, analgesics and antipyretics, non-steroidal anti-inflammatory and antirheumatic agents. Among the elderly who practiced self-medication, 55.5% used drugs that were inappropriate for the elderly, according to Beers criteria of 2015, and 56.9% used medications that showed therapeutic duplicity with the prescribed drugs. We identified 57 drugs used for self-medication, of which 30 (52.6%) were classified as over-the-counter and 27 (47.4%) as prescription drugs. Approximately 68.6% of elderly had at least one interaction involving drugs prescribed and those used for self-medication.

**Conclusion:**

The practice of self-medication was frequent in the elderly studied. The widespread use of over-the-counter drugs and/or potentially inappropriate medications for elderly increases the risk of drug interactions and adverse events.

## RESUMO

**Objetivo:**

Determinar o perfil dos medicamentos utilizados por automedicação por idosos.

**Métodos:**

Estudo transversal baseado em entrevistas com idosos atendidos de julho de 2014 a julho de 2015 em um centro de referência na Atenção à Saúde do Idoso de um hospital de ensino. Foram coletadas informações clínicas, demográficas e farmacoterápicas.

**Resultados:**

Entrevistaram-se 170 idosos, 85,9% eram mulheres e a mediana de idade foi 76 anos. A frequência de automedicação foi 80,5%. Os medicamentos mais utilizados por automedicação foram relaxantes musculares de ação central, analgésicos e antipiréticos, além dos anti-inflamatórios e antireumáticos não esteroidais. Entre os idosos que praticaram automedicação, 55,5% utilizaram medicamentos inapropriados para idosos, segundo os critérios de Beers de 2015, e 56,9% utilizam medicamentos que apresentavam duplicidade terapêutica com os medicamentos prescritos. Foram identificados 57 medicamentos utilizados por automedicação, e 30 (52,6%) eram classificados como isentos de prescrição e 27 (47,4%) como de venda sob prescrição médica. Cerca de 68,6% dos idosos apresentavam pelo menos uma interação envolvendo medicamentos prescritos e utilizados por automedicação.

**Conclusão:**

A prática de automedicação foi elevada nos idosos estudados. O amplo uso de medicamentos de venda livre e/ou potencialmente inapropriados para idosos aumenta o risco de interações medicamentosas e de eventos adversos.

## INTRODUCTION

The aging process of the population is associated to changes in the epidemiological profile of diseases, including an increase in degenerative chronic diseases, in the number of medications used, and in the demand for healthcare services.^(^ ^1^ ^)^ This evolution contributes to the lengthening of pharmacological treatment time and, therefore, the use of prescribed and non-prescribed medication.^(^ ^2^ ^)^

Self-medication is not universally defined. It can be described as the practice of selecting and using over-the-counter (OTC) medications, reusing previously prescribed medications with no healthcare professional supervision, and using prescription medication to treat self-diagnosed symptoms or diseases.^(^ ^3^ ^)^ Situations that can also be described as self-medication include: using medication recommended by friends or family members, non-adherence to a treatment plan, or changing the dose of prescribed medications.^(^ ^4^ ^)^

Self-medication requires special attention in the elderly. It presents an increased risk of drug interactions with a possible increase of adverse drug reaction (ADR), which can cause harm to patients, especially due to typical alterations brought by the aging process.^(^ ^1^ ^)^ There is also the risk of a late or incorrect diagnosis and prolonging the suffering associated to a disease.^(^ ^3^ ^)^ Another consequence of self-medication is increased resistance from the inadequate use of antimicrobials.^(^ ^5^ ^)^

## OBJECTIVE

To determine the profile of drugs used by the elderly population for self-medication.

## METHODS

This is a descriptive cross-sectional study carried out at a reference center for Elderly Health of a public teaching hospital, credentialed by the Brazilian Unified Healthcare System (SUS) [*Sistema Único de Saúde*], located in the city of Belo Horizonte (MG).

The study population was made up of elderly patients, here defined as ≥60 years, referred to pharmacist consultation by a multidisciplinary team.

We used a non-probabilistic sample with patients seen by the pharmacist between July 2014 and July 2015, and who met the inclusion criteria: individuals aged ≥60 years and on one or more medications.

Study variables were: self-medication; sex; drug interaction; therapeutic duplicity; use of medications included in the Beers List (2015 version)^(^ ^6^ ^)^ as potentially inappropriate for the elderly; dizziness; independency level for Activities of Daily Living (ADL) (dependent or independent); cognition (preserved or not-preserved); age; and number of prescribed medications.

Self-medication was defined as the use of OTC medication; reuse of previously prescribed medication with no healthcare professional supervision; and the use of prescription medication.

The drugs used by self-medication were classified according to the chemical subgroup (level 3) as described by the Anatomical Therapeutic Chemical Classification System (ATC) from the World Health Organization (WHO).^(^ ^7^ ^)^ We also verified if the drugs were included in OTC medication List of the Brazilian National Health Surveillance Agency (ANVISA),^(^ ^8^ ^)^ and in the list of potentially inappropriate drugs for elderly patients.^(^ ^6^ ^)^ We checked for therapeutic duplicity, *i.e*. the use of at least two drugs for the same indication, considering self-medication.

For this study, polypharmacy was defined as the simultaneous use of five or more medications. Drug interactions involving self-medication were identified through the software DRUG-REAX^®^ System by Truven Health Products.^(^ ^9^ ^)^

Data about cognition and ADL were collected from the patients’ medical charts. Independence levels for basic ADL were assessed by the Katz Index, which classified elderly patients as: Independent, when all basic living activities are performed with no help; Semi-dependent, when there is compromise of one of the functions (showering and/or getting dressed and/or using the bathroom); Incomplete dependent, if they there is compromise of a simple vegetative function (transference and/or continence) in addition to a dependency for showering, getting dressed and using the bathroom; and Complete Dependent, when all ADL are compromised, including eating. Instrumental ADLs were evaluated by the Lawton-Brody scale, and were classified as: Independent, when all instrumental activities of daily living are performed with no help; Partially dependent, when the individual can perform some tasks; and Completely dependent when the individual depends on others for all instrumental ADL. In this study, elderly individuals who presented some degree of dependency on both scales, either partial or total, were considered dependent. The Mini-Mental State Examination (MMSE), in combination with limitations or restrictions for ADL, was used to evaluate the presence of cognitive *deficit*. The cut-off point for low level of education/illiteracy was <18 points, and for high level of education, <26 points, of a total of 30 points.^(^ ^10^ ^)^

The aged individuals were referred by Primary Care professionals to the reference center, which was the setting of our investigation. Pharmacist consultation was given when requested by the team. The interview with the patients who met the inclusion criteria was done by the pharmacist, between July 2014 and July 2015.

The participants were interviewed, and the information was recorded on a data collection tool developed for the investigation, which encompasses clinical, demographic, and pharmacotherapeutic variables.

The collected data was digitized into a database created with the software EpiData 3.1. The descriptive analysis of the data was performed by determining the frequencies for categorical variables, and for continuous variables we used measures of central tendency (mean and median), and dispersion measures (standard deviation – SD - and interquartile range – IQR). Statistical analysis was performed using the software Statistical Package for Social Sciences (SPSS), version 21.0.

The study was approved by the Research Ethics Committee of *Universidade Federal de Minas Gerais* (COEP-MG), under CAAE: 58965316.6.0000.5149, and was developed according to all constant ethical principles of resolution 466/12 about research including humans. Patients’ identities were kept confidential.

## RESULTS

A total of 170 patients were included, most of whom were female (85.9%). Median age was 76 years (IQR=12). Regarding functionality, 60.6% of participants were dependent for instrumental ADL and 87.9% were independent for basic ADL. Cognition was found not to be preserved in 51.5%.

Polypharmacy was identified in pharmacotherapy of 165 (97.1%) of participants. The median number of medications per patient was 11 (IQR=5). We found that 80.6% of participants used self-medicated drugs, with a median of two drugs per participant (IQR=2). Of the 137 self-medicated patients, 76 (55.5%) used drugs included on the list of potentially inappropriate medications for the elderly, and 78 (56.9%) used drugs that presented therapeutic duplicity with their prescribed medications (Table 1).

**Table 1:** Clinical, functional and medication-use-related characteristics of the elderly participants

Variable	n (%)
Self-medication	
Yes	137 (80.6)
No	33 (19.4)
Drug interaction*	
Yes	94 (68.6)
No	43 (31.4)
Therapeutic duplicity including self-medication*	
Yes	78 (56.9)
No	59 (43.1)
Self-medication with drugs included in the Beers list 2015*	
Yes	76 (55.5)
No	61 (44.5)
Self-medication with prescription drugs*	
Yes	50 (36.5)
No	87 (63.5)
Basic ADL*	
Independent	145 (87.9)
Dependent	20 (12.1)
Instrumental ADL*	
Independent	65 (39.4)
Dependent	100 (60.6)
Cognition*	
Not preserved	85 (51.5)
Preserved	80 (48.5)
Polypharmacy	
Yes	165 (97.1)
No	5 (2.9)

^*^ The total varied according to the ignored information. AVD: Activities of Daily Living.

We identified 57 drugs used by self-medication – 30 of which (52.6%) were OTC, and 27 (47.4%) were prescription medications. Table 2 shows the drugs patients used without medical prescription, according to the ATC classification system level 3. Musculoskeletal drugs, including muscle-relaxants and nonsteroidal anti-inflammatory drugs (NSAIDs) were the most frequent, accounting for 36.1% of self-medicated drugs – 21.4% and 14.7%, respectively. They were followed by medications for the nervous system (35.3%) and the main pharmacological group of this category were the other antipyretics and analgesics, such as paracetamol and dipyrone. Drugs for the digestive tract and the metabolism accounted to 12.8% of self-medicated drugs and comprised several pharmacological groups. The most frequent were antacids, drugs for peptic ulcer, gastroesophageal reflux disease and constipation, and multivitamins.

**Table 2:** Distribution of self-medicated drugs used by the elderly patients according to level 3 of the Anatomical Therapeutic Chemical classification system

Anatomical Therapeutic Chemical Classification	n (%)
1. Musculoskeletal system	96 (36.1)
M03B – Central-acting muscle relaxants: dipyrone + orphenadrine + caffeine, caffeine + carisoprodol + diclofenac + paracetamol	57 (21.4)
M01A – Non-steroid anti-inflammatory and antirheumatic agents: diclofenac, phenylbutazone, ibuprofen, nimesulide, glucosamine sulfate + sodium chondroitin sulfate, meloxicam, lornoxicam	39 (14.7)
2. Nervous system	94 (35.3)
N02B – Other analgesics and antipyretics: dipyrone, paracetamol	83 (31.2)
N06B – Psychostimulants: dipyrone + caffeine + isometheptene	5 (1.9)
N07C – Antivertigo preparations: flunarizine	4 (1.5)
N02A – Opioids: tramadol + paracetamol	2 (0.8)
3. Digestive tract and metabolism	34 (12.8)
A02A – Antacids: sodium bicarbonate, magnesium hydroxide	7 (2.6)
A02B – Drugs for peptic ulcer and gastroesophageal reflux disease: omeprazole, cimetidine	6 (2.3)
A06A – Drugs for constipation: sodium picosulphate + *Senna alexandrina + Polygonum punctatum* + *Collinsonia canadensis,* bisacodyl, magnesium sulphate	5 (1.9)
A11A – Multivitamins, associations: vitamin complexes	5 (1.9)
A12A – Calcium	3 (1.1)
A03B – Belladonna e derivatives: scopolamine butylbromide	3 (1.1)
A07D – Antipropulsives: loperamide	2 (0.8)
A12C – Other mineral supplements	1 (0.4)
A03D – Antispasmodics in combination with analgesics	1 (0.4)
A11D – Vitamin B1	1 (0.4)
4. Respiratory system	8 (3.8
R05C – Expectorants, except for combinations with cough suppressors: ambroxol	1 (0.4)
R06A – Antihistamines of systemic use: buclizine, loratadine, promethazine, dexchlorpheniramine, paracetamol + chlorpheniramine + phenylephrine	7 (2.6)
5. Blood and blood forming organs	6 (2.3)
B01A – Antithrombotic agents: acetylsalicylic acid	5 (1.9)
B03A – Iron preparations: ferrous sulphate	1 (0.4)
6. Cardiovascular system	6 (2.3)
C10A – Lipid modifying agents: omega 3	2 (0.8)
C03A – Low-ceiling diuretics, thiazides: hydrochlorothiazide	1 (0.4)
C03D – Potassium sparing agents: spironolactone	1 (0.4)
C09A – Inhibitors of angiotensin-converting enzyme: captopril	1 (0.4)
C09C – Angiotensin II antagonists: losartan	1 (0.4)
7. Dermatological drugs	2 (0.8)
D06A – Antibiotic for topical use: mupirocin	1 (0.4)
D07A – Corticoids: dexamethasone	1 (0.4)
8. Anti-infectives for systemic use	2 (0.8)
J01X – Other antibacterial agents: acriflavine hydrochloride + methenamine + methylthioninium chloride + *Atropa belladonna* L	1 (0.4)
J02A – Antimycotics for systemic use: ketoconazole	1 (0.4)
9. Genitourinary system and sex hormones	1 (0.4)
G04B – Urinary system: sildenafil	1 (0.4)
10. Systemic hormonal drugs, except for sex hormones and insulins	1 (0.4)
H02A – Systemic corticoids: prednisone	1 (0.4)
Drugs not included on the ATC list	16 (6.0)
Total	266 (100)

ATC: Anatomical Therapeutic Chemical.

Regarding drug interactions, 94 (68.6%) participants presented at least one interaction involving both prescribed and self-medicated drugs. The median of interactions per participant was 1 (IQR=3). Table 3 shows the most frequent drug interactions and their respective severity and clinical effect. Of the 114 interactions detected, NSAIDs presented the most interactions - they were involved in 99 (86.6%) of all drug interactions identified.

**Table 3:** Drug interaction with absolute frequency above 5

Interaction	Severity	Clinical effect and action mechanism	n
ASA + dipyrone	Severe	Reduces ASA effectiveness by attenuating its antiplatelet effect	48
ASA + diclofenac	Severe	Increases risk of bleeding due to the additive effect on homeostasis	12
HCTZ + diclofenac	Severe	Reduces diuretic effects and may cause nephrotoxicity by reducing the production of renal prostaglandins	9
ASA + ibuprofen	Severe	Reduces ASA antiplatelet effect by competing for the COX-1 binding site and increases risk of bleeding by additive effect	8
Losartan + diclofenac	Moderate	Alters renal functions and/or increases BP by additive effect in the renal function and/or reduced the production of renal prostaglandins	8
Ibuprofen + diclofenac	Severe	Increases risk of bleeding by additive effect on homeostasis	7
ASA + nimesulide	Severe	Increases risk of bleeding by additive effect on homeostasis	6
Fluoxetine + diclofenac	Severe	Increases risk of bleeding by depleting the serotonin of platelets and by additive effect	6
Furosemide + ibuprofen	Severe	Reduces diuretic effectiveness and may cause nephrotoxicity by reducing the production of renal prostaglandins	6
Paracetamol + warfarin	Moderate	Increases risk of bleeding by inhibiting warfarin metabolism, or interfering in the formation of clotting factors	6
Furosemide + diclofenac	Severe	Reduces diuretic effectiveness and may cause nephrotoxicity by reducing the production of renal prostaglandins	6
HCTZ + nimesulide	Severe	Reduces diuretic effectiveness and may cause nephrotoxicity by reducing the production of renal prostaglandins	6
Enalapril + diclofenac	Severe	Alters renal functions and/or increases BP by additive effect on renal function and/or reduces the production of renal prostaglandins	6

ASA: acetylsalicylic acid; HCTZ: Hydrochlorothiazide; COX-1: cyclooxygenase 1; BP: blood pressure.

## DISCUSSION

The prevalence of elderly individuals who reported self-medication was quite high. This finding is in line with international^(^ ^11^ ^,^ ^12^ ^)^ and national^(^ ^13^ ^)^ studies that describe the prevalence of self-medication in the elderly as being above 70%. In Brazil, the prevalence of self-medication among the elderly is asymmetrical, varying between 8.9% and 80.5%.^(^ ^13^ ^-^ ^16^ ^)^ A systematic review about self-medication also showed this asymmetry, with prevalence rates varying between 4% and 87%.^(^ ^2^ ^)^ This asymmetry can be explained by factor variability, such as the different sociodemographic profiles of aged individuals included in the study, the adopted self-medication criteria, category of the described medication and the duration of self-medication.^(^ ^2^ ^,^ ^12^ ^)^

The most popular drugs among the elderly were those for the musculoskeletal system, which include central action muscle relaxants and NSAIDs. These pharmacological groups are described as frequently used by self-medication by adults and elderly individuals.^(^ ^1^ ^,^ ^5^ ^,^ ^17^ ^,^ ^18^ ^)^

Central action muscle relaxants are used to reduce and relieve symptoms of painful muscle spasms or the spasticity brought by musculoskeletal and neuromuscular disorders, both of which often affect the elderly and influence the practice of self-medication.^(^ ^19^ ^)^ However, these drugs may induce anticholinergic effects, sedation and increase the risk of fractures,^(^ ^6^ ^)^ which is dangerous to the elderly. Moreover, most muscle relaxants available in Brazil are present in fixed-dose combinations – often with NSAIDs. There is no solid scientific evidence that supports the use of these combinations, which further increases the risks of using these drugs by self-medication.^(^ ^19^ ^)^

Nonsteroidal anti-inflammatory drugs are also a widely used drug class for self-medication, especially for pain relief,^(^ ^20^ ^)^ but they are not recommended as a first line for chronic pain treatment due to the potential risk of gastrointestinal bleeding or peptic ulcer in high risk groups (age ≥75 years, use of oral or parenteral corticosteroids, anticoagulants or antiplatelet agents).^(^ ^6^ ^,^ ^20^ ^)^ There are also reports of nephrotoxicity associated to the use of NSAIDs. The use of NSAIDs without previous evaluation by a healthcare professional and with the presence of risk factors for these adverse events increases the chance of these events actually occurring and shows the danger of self-medicating with these drugs.^(^ ^21^ ^)^

One of the consequences of self-medicating is drug interaction. In this study, NSAIDs were the drug class most often involved in these interactions. Of the 11 most frequent interactions with NSAIDs, five increase the risk of bleeding by additive effect. A study evaluating ADR by self-medication found that NSAIDs were the drugs with more notifications and were associated to gastrointestinal pain and hemorrhage.^(^ ^18^ ^)^

Among the drugs that act on the nervous system, analgesics were the most often taken by self-medication. Although dipyrone and paracetamol are considered safe for the elderly, they are not free of risk when used indiscriminately and without the instruction of a healthcare professional.^(^ ^17^ ^)^ Analgesics, antipyretics, and non-opioid antirheumatics are related to 37% of hospital admissions among the elderly for poisoning and ADR in Brazil.^(^ ^22^ ^)^

The interaction between dipyrone and acetylsalicylic acid (ASA) was the most frequent, and it may reduce ASA effectiveness and expose the patient to cardiovascular events when dipyrone is used for at least three consecutive days in doses of 1,500 to 4,000mg per day.^(^ ^9^ ^)^ The prolonged use of flunarizine induces parkinsonism,^(^ ^23^ ^)^ and elderly patients should be advised not to use it indiscriminately without first consulting a physician.

The use of omeprazole and cimetidine by elderly patients by self-medication is worrisome. Cimetidine inhibits oxidative metabolism and increases the half-life of several drugs, thus increasing the risk of drug interactions and adverse reactions.^(^ ^10^ ^)^ The use of omeprazole for more than 8 weeks should be avoided, except in high-risk patients, because it increases the possibility of infections by *Clostridium difficile*, bone loss, and fractures.^(^ ^6^ ^)^

One adverse event that may be caused by medication in the elderly is falls. Drugs used by self-medication can significantly contribute to falls due to their adverse effects, such as compromised balance and coordination, mental confusion, cognitive deficit, sedation and orthostatic hypotension, thus increasing the risk of fractures and reducing quality of life.^(^ ^24^ ^,^ ^25^ ^)^

The elevated use of potentially inadequate drugs by self-medication is alarming because the adverse effects outweigh the benefits. It is important to avoid using these drugs by self-medication to maintain the quality of life of elderly individuals, preserve their functionality, and reduce the risk of adverse effects.^(^ ^26^ ^,^ ^27^ ^)^

In relation to health regulation, most of the drugs used by self-medication were classified as OTC. We also identified prescription drugs and controlled prescription drugs, which is alarming because these drugs can be harmful without previous medical assessment. Even though most of the drugs were OTC, they still pose risks of adverse events.

Another aspect that favors and influences elderly patients to self-medicate is advertising pieces in the media put out by the pharmaceutical industry.^(^ ^17^ ^)^ These ads only describe benefits and there is no clarification as to the associated risks, thus giving the general population the idea that the product is risk-free.^(^ ^28^ ^)^ Furthermore, patient-oriented medication package inserts registered with ANVISA do not provide information that guarantees a safe use by elderly.^(^ ^19^ ^)^

Strategies and interventions to limit the improper use of medications must be adopted. Healthcare professionals can promote the rational use of medications as a strategy to educate the population, and, therefore, reduce the problems related to the use of medications without orientation.

This study presents a broad approach of the use of drug by self-medication by elderly individuals, and considers some pharmacotherapeutic aspects, such as drug interactions and medications that are potentially inadequate for elderly individuals – which have not been evaluated in depth in previous studies. Therefore, this study provides important contributions for clinical practice and suggests methods to prevent self-medication among elderly patients.

Some limitations must be considered. Firstly, we did not work with a probabilistic sample, the great majority of our participants were female, and we worked with only one reference center. These factors keep our results from being extrapolated to all elderly individuals. Secondly, the period of self-medication was not defined before the interview, which may have contributed to maximize the frequency of drug use by self-medication.

## CONCLUSION

The rate of medications used by the elderly population without first consulting a healthcare professional was proven to be high. Central action muscle relaxants, analgesics and antipyretics, and non-steroidal anti-inflammatory and antirheumatic agents were the most often self-medicated drugs among the elderly. The wide use of Over-the-counter and/or potentially inappropriate drugs increases the risk of drug interactions, which may cause adverse events.
